# Telephone-Based Intervention to Improve Family Planning Care in Pregnancies of Unknown Location: Retrospective Pre-Post Study

**DOI:** 10.2196/42559

**Published:** 2023-08-28

**Authors:** Anne Nichols Flynn, Nathanael C Koelper, Sarita Sonalkar

**Affiliations:** 1 University of California, Davis Sacramento, CA United States; 2 Department of Obstetrics and Gynecology University of Pennsylvania Philadelphia, PA United States

**Keywords:** contraception, electronic medical record, family planning, pregnancy intendedness, pregnancy of unknown location, pre-post study, telephone-based intervention

## Abstract

**Background:**

Patients followed for a pregnancy of unknown location are generally followed by a team of clinicians through telephone calls, and their contraceptive needs at the time of pregnancy resolution may not be addressed.

**Objective:**

This study aimed to assess contraceptive counseling and contraceptive uptake before and after a telephone-based intervention.

**Methods:**

This was a retrospective pre-post study assessing pregnancy intendedness in patients with a pregnancy of unknown location and the proportion of patients who received contraceptive counseling and a contraceptive prescription before and after the initiation of a telephone-based intervention. We reviewed medical records 1 year before and 1 year after implementation of our intervention for demographic characteristics, pregnancy intendedness, pregnancy outcome, contraceptive counseling documentation, receipt of contraception, and repeat pregnancy within 6 months. We assessed the effects of an implementation strategy to address family planning needs once pregnancy was resolved by comparing the proportions of patients who were counseled and received contraception before and after our intervention was implemented. We performed logistic regression to identify associations between covariates and the outcomes of contraceptive counseling documentation and receipt of contraception.

**Results:**

Of the 220 patients in the combined cohort, the majority were Black (161/220, 73%) and ultimately had a resolved pregnancy of unknown location (162/220, 74%), and the proportion of pregnancies documented as unintended was 60% (132/220). Before our intervention, 27 of 100 (27%) patients received contraceptive counseling, compared with 94 of 120 (78%) patients after the intervention (odds ratio [OR] 9.77, 95% CI 5.26-18.16). Before the intervention, 17 of 90 (19%) patients who did not desire repeat pregnancy received contraception, compared with 32 of 86 (37%) patients after the intervention (OR 2.54, 95% CI 1.28-5.05). Our postintervention cohort had an increased odds of receiving contraceptive counseling (OR 9.77, 95% CI 5.26-18.16) and of receiving a contraceptive prescription (OR 2.54, 95% CI 1.28-5.05) compared with our preintervention cohort.

**Conclusions:**

We found that over half of patients with a pregnancy of unknown location have an unintended pregnancy, and standardization of care through a telephone-based intervention improves contraceptive counseling and prescribing in patients with a resolved pregnancy of unknown location. This intervention could be used at any institution that follows patients with a pregnancy of unknown location remotely to improve care.

## Introduction

In up to 40% of pregnant patients, ultrasound does not definitively identify a pregnancy location, a transient state called pregnancy of unknown location [1,2]. Follow-up for patients with a pregnancy of unknown location often includes serial human chorionic gonadotropin (hCG) measurements with telephone calls to determine next steps and ultimately determine a final diagnosis. While private obstetrics and gynecology physicians may follow these patients individually, at academic institutions, these patients are generally followed through a patient list by 1 or a team of obstetrics and gynecology residents or clinicians.

For patients undergoing serial hCG measurements, a substantial portion of care is conducted through laboratories and telephone calls rather than in office care; if hCG resolves spontaneously, many patients do not have a visit with a health care clinician after an initial consult or emergency department visit and thus do not get a chance to ask questions or have health needs addressed, such as contraception [1]. Most often, contraceptive counseling occurs in a clinician’s office either at the time of a contraceptive consultation, during an annual well-person visit, at a postpartum visit, during management of early pregnancy loss, or management of an undesired pregnancy. Contraceptive counseling involves a query regarding fertility desires and, if pregnancy prevention is valued, a discussion regarding methods of contraception, risks, side effects, and facilitation of contraceptive method access either through prescription or recommendation for an office visit.

Patients in the postpartum or postabortion period who are also experiencing recent pregnancy are often able to undergo contraceptive counseling in person after resolution of their pregnancy. For postpartum patients, contraception counseling and provision can occur in the hospital, and for patients seeking induced abortion or management of pregnancy loss, contraceptive counseling provision often occurs immediately after management of the pregnancy. However, patients with a resolved pregnancy of unknown location are often discharged from care by a telephone call reporting an undetectable hCG. Discussing contraception and future pregnancy planning over the phone is possible but not standardized, particularly if these patients are managed as part of team-based care. Given that patients with a pregnancy of unknown location are often managed remotely, they present a unique challenge for addressing these important family planning needs. Furthermore, this patient population who recently experienced a pregnancy loss may wish to initiate contraception either because they had an unplanned pregnancy or to allow for time to emotionally recover from their pregnancy loss [3].

In this study, our objectives were as follows: (1) to determine the proportion of patients with a pregnancy of unknown location who classify their pregnancy as unintended and (2) to determine the reach and effectiveness of a simple, reproducible, telephone-based intervention to improve contraceptive counseling once a pregnancy of unknown location is resolved.

## Methods

### Study Design and Setting

This was a retrospective cohort study of patients with a resolved pregnancy initially presenting with a pregnancy of unknown location. We performed a retrospective chart review from October 2016 to October 2018, which was performed by the primary author. Any discrepancies in the chart review were reviewed by the senior author. We initiated a telephone-based electronic medical record (EMR) intervention on October 31, 2017, to improve contraceptive counseling upon discharge from serial hCG monitoring [4,5]. Patients were included if (1) they presented to our institution’s emergency department or outpatient office and were initially classified as having a pregnancy of unknown location, and (2) they had documented resolution of a pregnancy of unknown location through diagnosis and treatment of early pregnancy loss, a resolved pregnancy of unknown location, or medical or surgical management of an ectopic pregnancy [1]. They were excluded if they were lost to follow-up (defined as 2 weeks without being able to reach the patient), if their last evaluation was in the office, or if they were diagnosed with a viable intrauterine pregnancy (IUP) (that they continued) or molar pregnancy.

### Telephone Intervention, Data Collection, and Analysis

**Figure 1:**
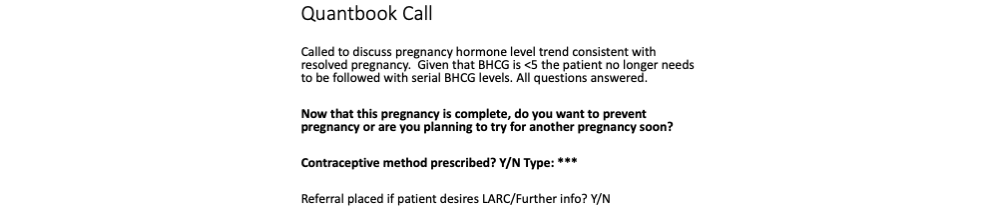
The electronic medical record script used to prompt residents to discuss future reproductive plans and contraception over the phone. BHCG: beta-human chorionic gonadotropin; LARC: long-acting reversible contraception.

Our intervention consisted of a standardized telephone script in the EMR upon discharge from hCG monitoring. This EMR script prompted clinicians to discuss the patients’ final diagnosis and reproductive plans (Figure 1). The telephone script first gave the clinicians a prompt for why they were calling and to explain that the pregnancy had resolved. It then prompted providers to ask, “Now that this pregnancy is complete, do you want to prevent pregnancy, or are you planning to try for another pregnancy soon?” If the patient was planning to prevent pregnancy, clinicians were prompted to discuss contraceptive options, prescribe methods, or refer for a contraception appointment as appropriate (Figure 1). We abstracted demographic characteristics, index pregnancy intention and desiredness (documented through EMR prompts in the initial consult note), documentation of contraceptive counseling after resolution of pregnancy, prescription of contraception, and repeat pregnancy within 6 months (if available within the EMR). We compared contraceptive counseling documentation in a cohort of patients before the introduction of the EMR intervention and in a cohort of patients after the intervention to assess the penetration of the intervention and contraceptive prescriptions for those who wished to prevent pregnancy before and after the intervention to assess the effectiveness of the intervention. Contraceptive counseling was defined as any documented discussion in the EMR of contraception at the time of discharge from follow-up; this included any referrals for outpatient contraceptive discussion. Through the chart review, we assessed contraceptive prescriptions by looking within the documentation of the telephone call, any associated orders with the call, or any long-acting reversible contraception initiated due to a referral. We performed descriptive and bivariate statistics, including the chi-square test for categorical data and Student’s *t* test or Wilcoxon rank sum test where appropriate for continuous data. We performed logistic regression to identify associations between covariates and the outcomes of contraceptive counseling documentation and receipt of contraception. The following covariates were assessed for inclusion in our final model with backward stepwise selection: age, race, parity, pregnancy intendedness, resident level, quarter of the academic year, final diagnosis, and type of treatment received to resolve the pregnancy. These covariates were chosen as potential variables that may affect provider assumptions regarding the need for contraceptive counseling as well as provider experience level. All data were analyzed using STATA (version 14.2; StataCorp).

### Ethical Approval

This study received institutional review board approval from the University of Pennsylvania institutional review board (protocol # 829436). A waiver of informed consent was granted given that the research was no more than minimal risk and could not have been practically carried out without the waiver.

## Results

### Population and Pregnancy Intention

Over the 2-year time period, 381 women underwent follow-up for a pregnancy of unknown location during the preintervention cohort and 320 underwent follow-up in the postintervention cohort. A total of 100 patients in our preintervention cohort and 120 in our postintervention cohort met our inclusion and exclusion criteria. These groups did not differ statistically in their baseline characteristics (Table 1). The majority of our combined cohort population was Black (161/220, 73%), presenting initially with vaginal bleeding (157/220, 71%), and ultimately had a resolved pregnancy of unknown location (162/220, 74%). In the combined cohort, 60% (132/220) of patients reported that their pregnancy was unplanned, 33% (73/220) reported that it was planned, and 7% (15/220) were uncertain or had an unknown pregnancy intention. Approximately 70% (155/220) reported that the pregnancy was desired in the combined cohorts (Table 1). Of all the patients enrolled in follow-up for a pregnancy of unknown location, the loss to follow-up rate in the preintervention time period was 24.3% (92/379) and 20.2% (64/317) in the postintervention time period.

**Table 1:** Baseline characteristics of the patients before and after initiation of a telephone-based contraceptive counseling intervention in patients with a resolved pregnancy of unknown location.

Characteristic		Preintervention, (n=100)	Postintervention, (n=120)	*P* value	
Age (years), mean (range)		28 (26-35)	29 (25-34)	.90	
**Race, n (%)**					.55
	Asian	7 (7)	9 (8)		
	Black	72 (72)	8 (74)		
	White	12 (12)	17 (14)		
	Other	9 (9)	5 (4)		
**Parity, n (%)**					.68
	0	38 (38)	52 (43)		
	1	26 (26)	27 (23)		
	2	23 (23)	23 (19)		
	3	6 (6)	5 (4)		
	4+	7 (7)	13 (11)		
**Chief complaint, n (%)**					.21
	Pain	20 (20)	16 (13)		
	Bleeding	71 (71)	86 (72)		
	Other or unknown	9 (9)	18 (15)		
**Final diagnosis, n (%)**					.99
	Ectopic	21 (21)	26 (22)		
	IUP^a^	5 (5)	6 (5)		
	Resolved pregnancy of unknown location	74 (74)	88 (73)		
**Pregnancy, n (%)**					.54
	Unplanned	63 (63)	69 (58)		
	Planned	32 (32)	41 (34)		
	Unknown	5 (5)	10 (8)		
**Desiredness, n (%)**					.35
	Undesired	16 (16)	18 (15)		
	Desired	68 (68)	87 (73)		
	Unknown	11 (11)	6 (5)		
	Uncertain	5 (5)	9 (8)		

^a^IUP: intrauterine pregnancy.

### Penetration and Effectiveness of the Intervention

Before the intervention, 27 of 100 patients (27%, 95% CI 19%-37%) received contraceptive counseling, compared with 94 of 120 patients (78%, 95% CI 70%-85%) after the initiation of the intervention, and 10 of 90 patients (19%, 95% CI 12%-28%) who wished to prevent pregnancy received contraception, compared with 32 of 86 patients (37%, 95% CI 28%-48%) after the intervention. A total of 10 patients in the preintervention cohort and 34 patients in the postintervention cohort were excluded from the effectiveness calculation due to documentation of a desire for repeat pregnancy. In the postintervention cohort, when contraceptive counseling was documented (n=94), the specified EMR telephone prompt intervention was used 79% (n=74; 95% CI 69%-86%) of the time. In the preintervention cohort, the 6-month repeat pregnancy rate was 26% (n=26). Of these 26 women, 80.8% (n=21) did not receive contraceptive counseling. In the second cohort, the 6-month repeat pregnancy rate was 26% (n=31), of whom 23% (n=7) did not receive contraceptive counseling.

We used logistic regression to assess whether any of the recorded covariates were associated with contraceptive counseling documentation and contraception initiation. Our postintervention cohort had an increased odds of receiving contraceptive counseling compared with our preintervention cohort (odds ratio [OR] 9.77, 95% CI 5.26-18.16). After adjusting for the quarter of the year and pregnancy intention, the postintervention cohort still had an increased odds of contraceptive counseling (adjusted OR 11.84, 95% CI 6.07-23.10; Table 2). Our postintervention cohort also had an increased odds of receiving a contraceptive prescription compared with our preintervention cohort (OR 2.54, 95% CI 1.28-5.05). After adjusting for pregnancy intention and treatment, the postintervention cohort still saw an increased odds of receiving a contraceptive prescription (adjusted OR 2.26, 95% CI 1.10-4.63; Table 3). Having a planned pregnancy was associated with 65% decreased odds of receiving a contraceptive prescription (OR 0.35, 95% CI 0.14-0.86).

**Table 2:** Logistic regression model for contraceptive counseling documentation before and after initiation of contraceptive counseling with a telephone-based intervention in patients with a resolved pregnancy of unknown location.

Variable			Odds ratio (95% CI)		Adjusted odds ratio (95% CI)		*P* value	
**Cohort**								<.01
	Pre (reference)	1		1		—^a^		
	Post	9.77 (5.26-18.16)		11.84 (6.07-23.10)		—		
**Quarter of year**								
	November-January (reference)	1		1		—		
	February-April	(0.48-2.06)		1.37 (0.57-3.28)		.48		
	May-July	0.85 (0.42-1.74)		0.9 (0.39-2.08)		.80		
	August-October	1.30 (0.61-2.8)		2.53 (0.99-6.46)		.05		
**Planned pregnancy status**								
	Unplanned (reference)	1		1		—		
	Planned	0.83 (0.46-1.46)		0.6 (0.3-1.2)		.30		
	Unknown or uncertain	0.87 (0.3-2.54)		0.45 (0.13-1.59)		.22		

^a^Not available.

**Table 3:** Logistic regression model for contraceptive initiation before and after initiation of contraceptive counseling with a telephone-based intervention in patients with a resolved pregnancy of unknown location.

Variable		Odds ratio (95% CI)	Adjusted odds ratio (95% CI)	*P* value
**Cohort**				.03
	Pre (reference)	1	1	—^a^
	Post	2.54 (1.28-5.05)	2.26 (1.10-4.63)	—
**Planned pregnancy status**				
	Unplanned (reference)	1	1	—
	Planned	0.36 (0.15-0.88)	0.35 (0.14-0.86)	.02
	Unknown or uncertain	2.16 (0.59-7.93)	2.12 (0.55-8.11)	.27
**Treatment**				
	Other (reference)	1	1	—
	Manual vacuum aspiration	2.34 (0.98-5.58)	2.14 (0.85-5.42)	.11

^a^Not available.

## Discussion

### Principal Findings

In our population, over half of patients with a pregnancy of unknown location reported having an unintended pregnancy. Before initiating an EMR intervention, contraceptive counseling at the time of discharge from monitoring was low, but with the use of a telephone-based intervention recorded in the EMR, the incidence of contraceptive counseling nearly tripled. We saw very high reach of the specific EMR telephone prompt by clinicians. The receipt of contraceptive prescriptions increased as well, but not proportionally.

### Comparison to Previous Work

To our knowledge, there is limited evidence regarding contraceptive counseling and contraceptive preferences in patients who are diagnosed with a resolved pregnancy of unknown location. Before using our EMR intervention, providers rarely discussed contraception with patients who had a resolved pregnancy of unknown location. In a qualitative study, investigators sought to explore clinicians’ views surrounding discussing contraception with patients after early pregnancy loss [6]. Within this study, they found that clinicians were reluctant to discuss contraception, and they also highlighted a barrier to providing contraceptive counseling as a lack of time [6]. This qualitative study highlights the possible bias providers experience surrounding patients experiencing pregnancy loss. In our study, the majority of patients had a resolved pregnancy of unknown location which is clinically similar to experiencing an early pregnancy loss. While our contraceptive counseling increased after the initiation of our EMR intervention, we did not see the same increase in contraceptive uptake. In a secondary analysis of a multicenter randomized controlled trial, investigators looked at contraceptive uptake in a patient population who had experienced an early pregnancy loss or miscarriage [3]. Within this population, roughly 50% (121/244) of them had planned pregnancies, which is higher than our cohort. They saw 40% (97/244) of participants initiated a form of contraception, compared with the 37% seen in our postintervention cohort [3]. This patient population was seen in clinic for counseling, and the slightly higher contraceptive uptake may highlight the difficulties inherent in contraceptive counseling and prescription for patients who are followed remotely, such as in pregnancies of unknown location. However, this may also highlight that not all patients are ready to initiate contraception immediately after experiencing a resolved pregnancy (miscarriage or resolved pregnancy of unknown location), regardless of their future intentions.

### Strengths and Limitations

We demonstrated that a simple, novel, and generalizable intervention can improve contraceptive counseling and uptake in patients with a resolved pregnancy after a diagnosis of a pregnancy of unknown location. In 2016, the National Quality Forum endorsed performance measures for contraceptive care. One of these measures was evaluating the percentage of people at risk for unintended pregnancy who are provided with a most or moderately effective contraceptive method [7]. In order to effectively use performance measures, it is necessary to create mechanisms for clinicians to adhere to them. For example, in order to successfully expand access to contraception, clinicians must do the following: address contraceptive needs, offer evidence-based contraceptive services, and provide patient-centered care [8]. This study used a simple initiative to improve contraceptive access for women at risk for pregnancy, a performance measure, while keeping patient preferences a priority and educating clinicians about the importance of assessing reproductive life planning at the point of care.

This study had a number of limitations. First, it was conducted as a retrospective prepost design, potentially affecting the internal validity. We therefore only had access to information that was charted, and we were unable to account for possible undocumented discussions between clinicians and patients regarding future fertility or desire for contraception. Second, only a single time point and brief prompts were used to assess pregnancy intention and desiredness; pregnancy desires are not necessarily dichotomous and can change over time [9,10]. Finally, although we tracked the provision of contraception, we did not follow up to confirm receipt of contraceptive prescriptions and long-term adherence. Although we saw a large increase in contraceptive counseling, contraceptive initiation increased to a smaller degree, and repeat pregnancy rates were not influenced. Due to the retrospective nature of this study, it is not clear if this was due to patient preferences or barriers to prescriptions and referrals in the case of long-acting reversible contraception. Future research should focus on optimizing follow-up for patients with a pregnancy of unknown location and streamlining access to outpatient offices for patients who wish to discuss contraception during a contraceptive visit or desire a long-acting reversible contraception.

### Conclusions

Patients presenting with a pregnancy of unknown location to the emergency department may report a high rate of unintended pregnancy. With the use of a telephone-based contraceptive counseling intervention documented in the EMR, the incidence of contraceptive counseling increased substantially. Our intervention is easily translatable to any institution with an EMR and a patient population that requires serial hCG monitoring, with follow-up conducted primarily remotely, for a pregnancy of unknown location.

## Abbreviations

EMR: electronic medical record
hCG: human chorionic gonadotropin
IUP: intrauterine pregnancy
OR: odds ratio

## Data Availability

The data set generated and analyzed during this study are available from the corresponding author on reasonable request.

## References

[1] Barnhart K, van Mello NM, Bourne T, Kirk E, Van Calster B, Bottomley C, Chung K, Condous G, Goldstein S, Hajenius PJ, Mol BW, Molinaro T, O'Brien KLO, Husicka R, Sammel M, Timmerman D (2011). Pregnancy of unknown location: a consensus statement of nomenclature, definitions, and outcome. Fertil Steril.

[2] Bobdiwala S, Al-Memar M, Farren J, Bourne T (2017). Factors to consider in pregnancy of unknown location. Womens Health (Lond).

[3] Roe AH, McAllister A, Sammel MD, Schreiber CA (2020). Pregnancy intentions and contraceptive uptake after miscarriage. Contraception.

[4] Powell BJ, McMillen JC, Proctor EK, Carpenter CR, Griffey RT, Bunger AC, Glass JE, York JL (2012). A compilation of strategies for implementing clinical innovations in health and mental health. Med Care Res Rev.

[5] Bauer MS, Damschroder L, Hagedorn H, Smith J, Kilbourne AM (2015). An introduction to implementation science for the non-specialist. BMC Psychol.

[6] Narayanan N, Reynolds-Wright JJ, Cameron ST (2022). Views of clinicians towards providing contraceptive advice and contraception to women following early pregnancy loss: a qualitative study. BMJ Sex Reprod Health.

[7] (2019). Performance measures. Office of Population Affairs.

[8] Moniz MH, Gavin LE, Dalton VK (2017). Performance measures for contraceptive care: a new tool to enhance access to contraception. Obstet Gynecol.

[9] Cutler A, McNamara B, Qasba N, Kennedy HP, Lundsberg L, Gariepy A (2018). "I just don't know": an exploration of women's ambivalence about a new pregnancy. Womens Health Issues.

[10] Santelli JS, Lindberg LD, Orr MG, Finer LB, Speizer I (2009). Toward a multidimensional measure of pregnancy intentions: evidence from the United States. Stud Fam Plann.

